# Supplementary material for: Identification of Markers Associated with Wheat Dwarf Virus (WDV) Tolerance/Resistance in Barley (Hordeum vulgare ssp. vulgare) Using Genome-Wide Association Studies
Source: Viruses. 2023 Jul 18;15(7):1568. doi: 10.3390/v15071568 (PMC10385604; doi:10.3390/v15071568)
Supplement: Supplementary file 1 [file viruses-15-01568-s001.zip › Figure S1 to S3.zip!/figure S1.pdf]

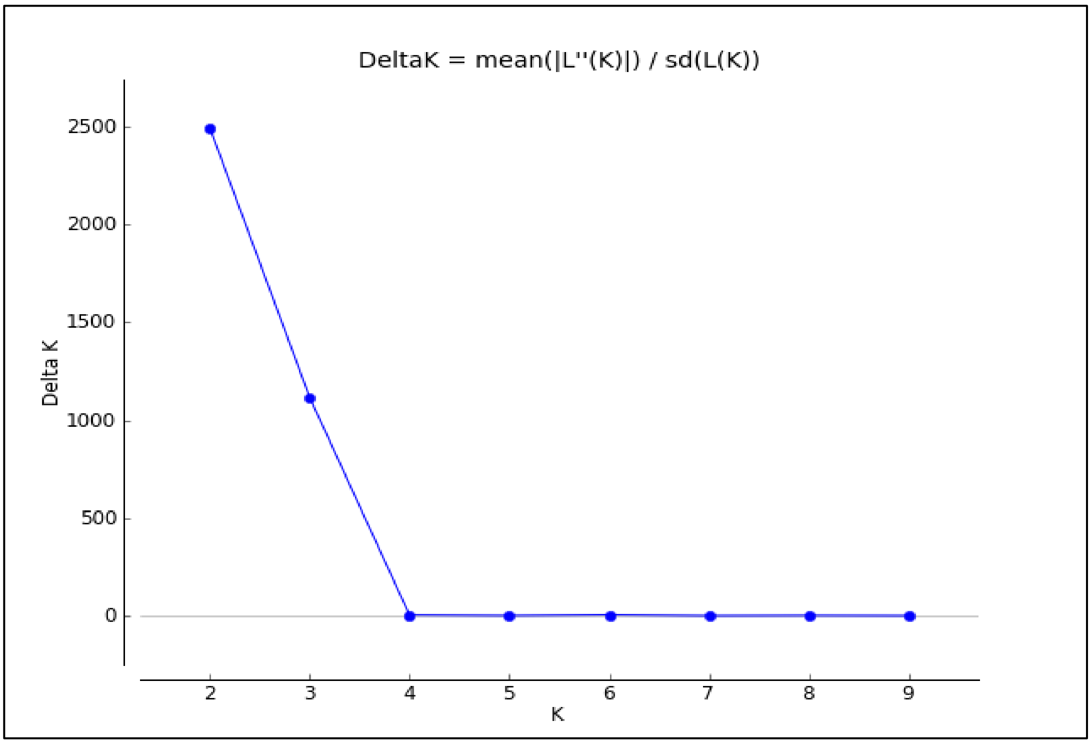

Figure S1: DeltaK plot: Number of optimal detected clusters (K= 3) according Bayesian clustering approach.
