# Supplementary material for: Identification of Markers Associated with Wheat Dwarf Virus (WDV) Tolerance/Resistance in Barley (Hordeum vulgare ssp. vulgare) Using Genome-Wide Association Studies
Source: Viruses. 2023 Jul 18;15(7):1568. doi: 10.3390/v15071568 (PMC10385604; doi:10.3390/v15071568)
Supplement: Supplementary file 1 [file viruses-15-01568-s001.zip › Figure S1 to S3.zip!/Figure S2.pdf]

(a)

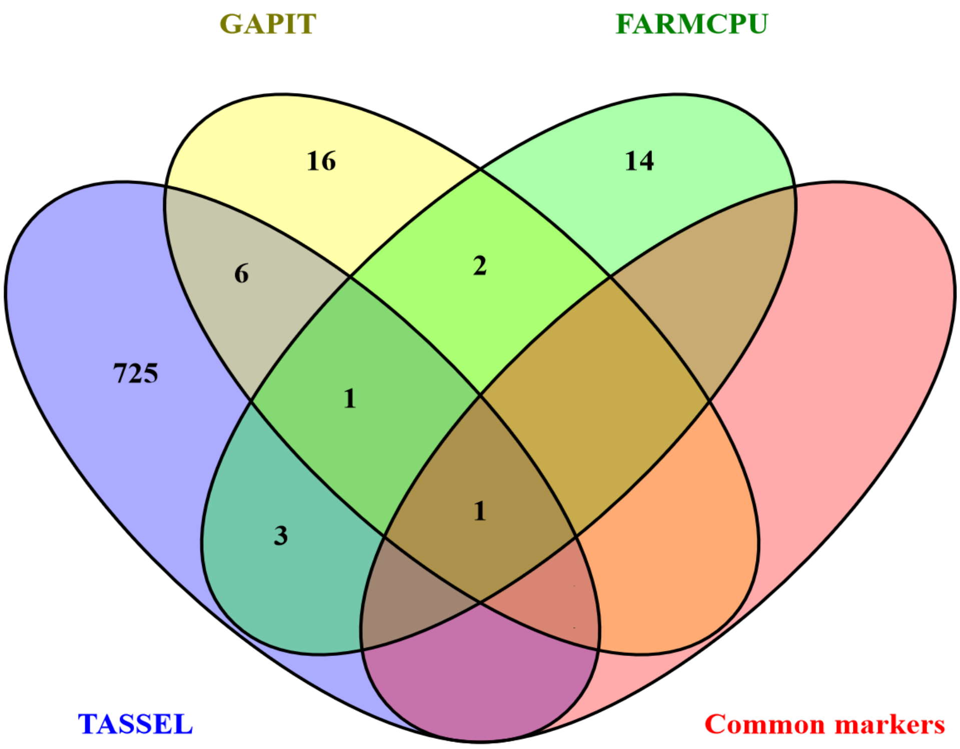
GAPIT
FARMCPU
16
14
6
2
725
1
1
3
TASSEL
Common markers

(b)

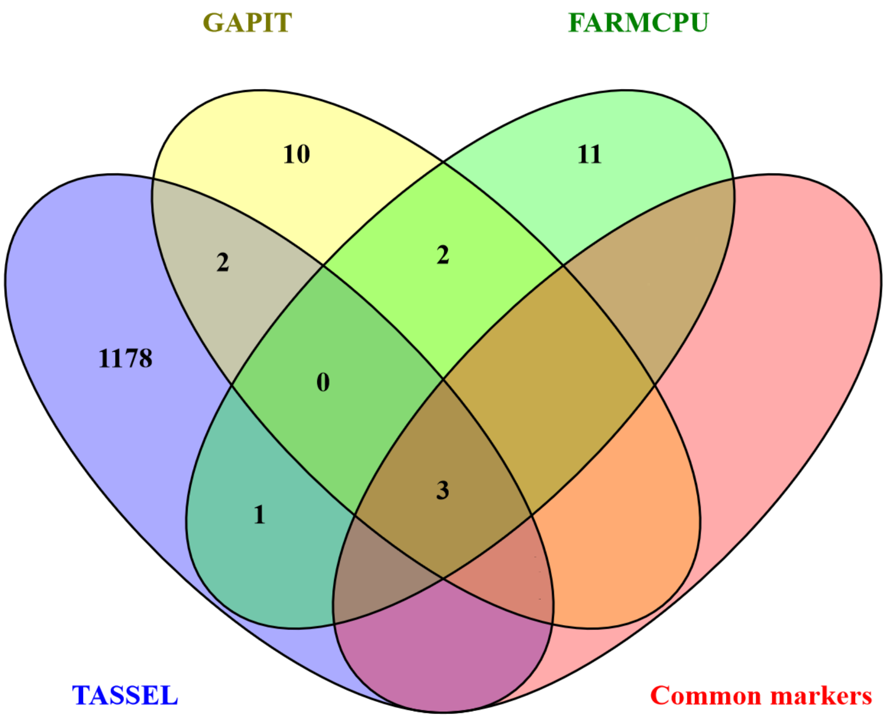
GAPIT
FARMCPU
10
11
2
2
1178
0
3
1
TASSEL
Common markers

(c)

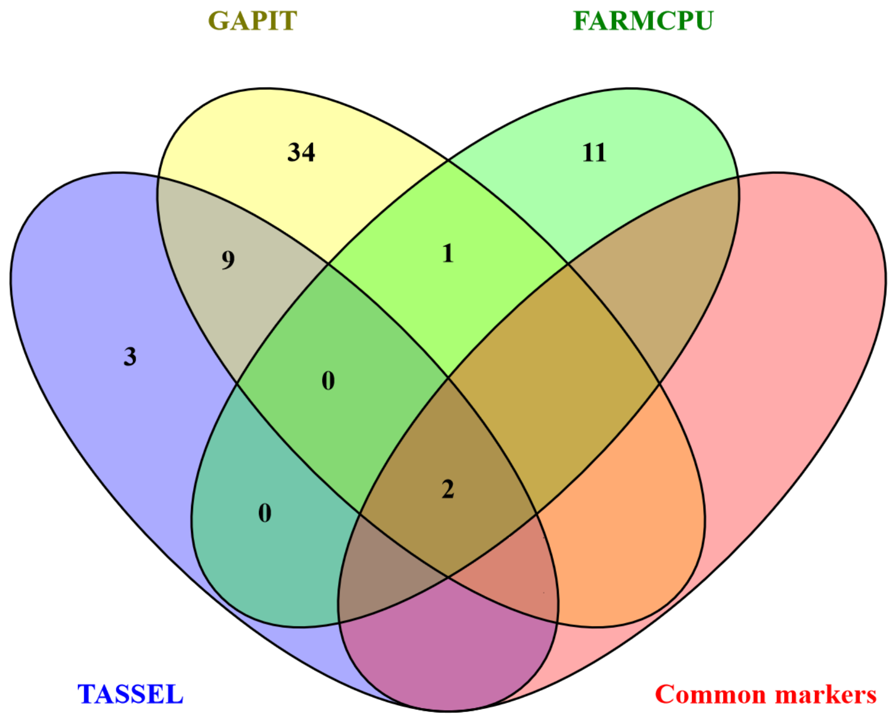
GAPIT
FARMCPU
34
11
9
1
3
0
2
0
TASSEL
Common markers

(d)

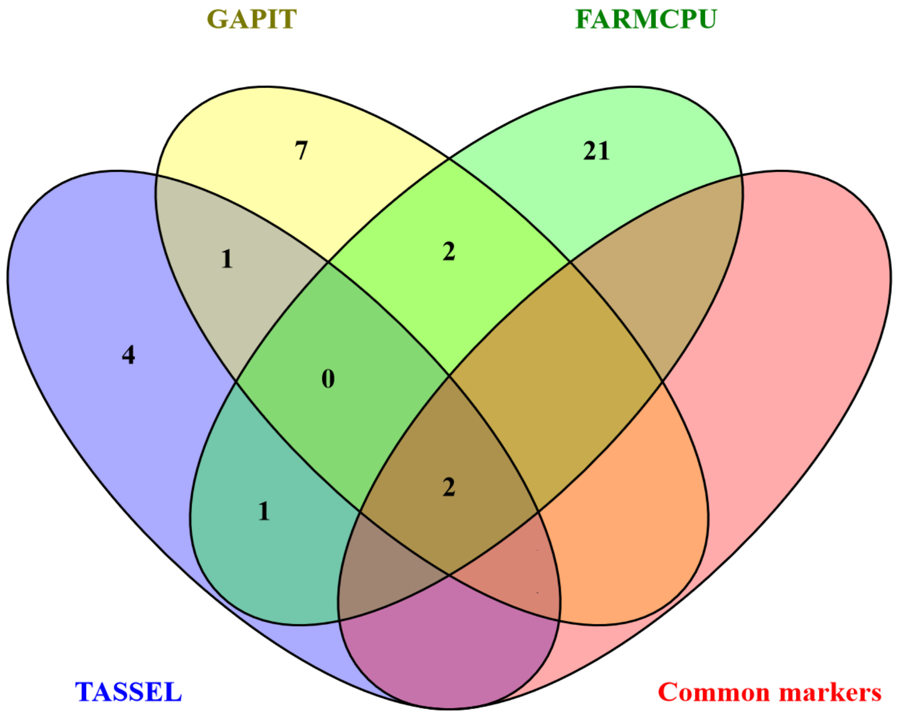
GAPIT
FARMCPU
7
21
1
2
4
0
2
1
TASSEL
Common markers

(e)

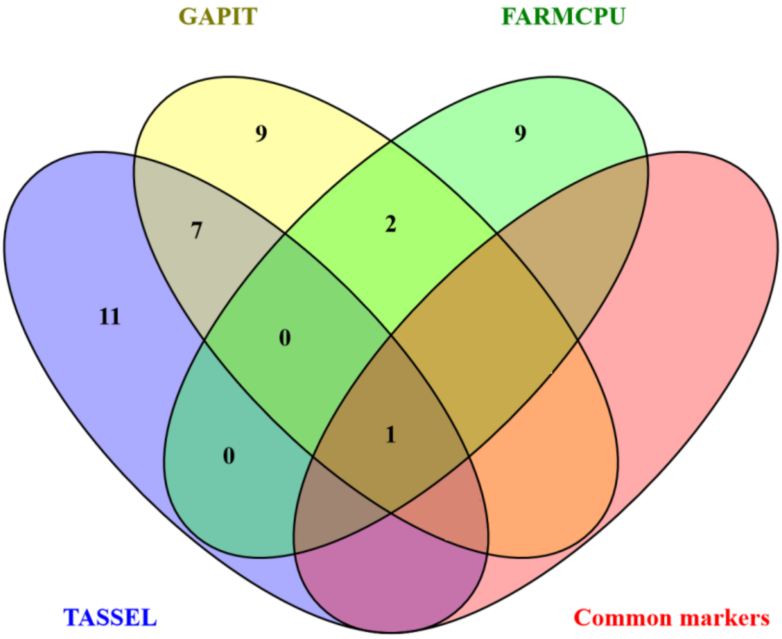

Figure S2: venn plot indicates number of identified markers (LOD≥3) with different methods for (a) relative virus titer, (b) relative total grain weight (c) relative plant height, (d) relative number of ears per plant, (e) relative thousand grain weight.
