# Supplementary material for: Identification of Markers Associated with Wheat Dwarf Virus (WDV) Tolerance/Resistance in Barley (Hordeum vulgare ssp. vulgare) Using Genome-Wide Association Studies
Source: Viruses. 2023 Jul 18;15(7):1568. doi: 10.3390/v15071568 (PMC10385604; doi:10.3390/v15071568)
Supplement: Supplementary file 1 [file viruses-15-01568-s001.zip › Figure S1 to S3.zip!/Figure S3.pdf]

(a)

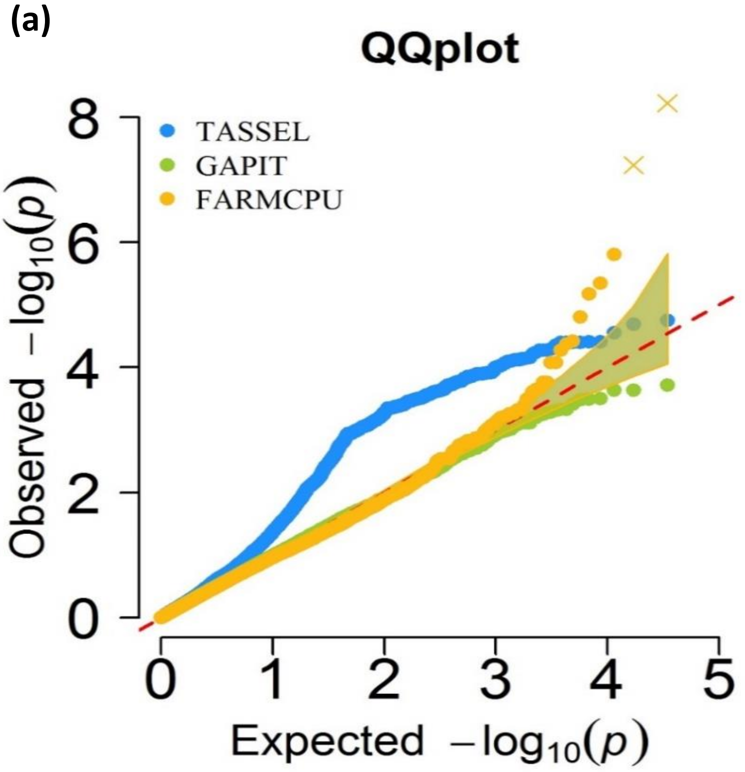

QQplot
TASSEL
GAPIT
FARMCPU
Observed $-\log_{10}(p)$
Expected $-\log_{10}(p)$
0
2
4
6
8
0
1
2
3
4
5

(b)

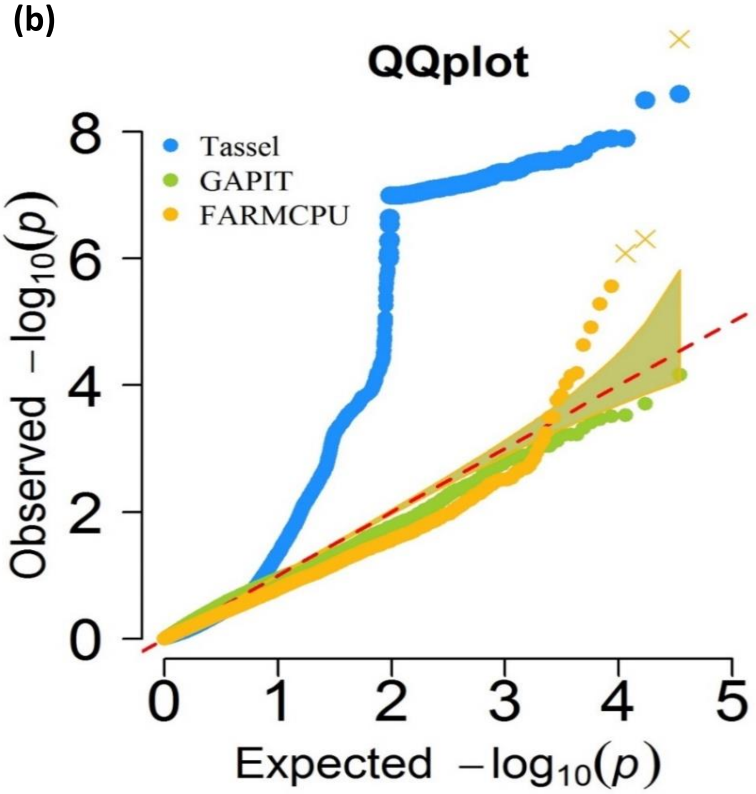

QQplot
Tassel
GAPIT
FARMCPU
Observed $-\log_{10}(p)$
Expected $-\log_{10}(p)$
0
2
4
6
8
0
1
2
3
4
5

(c)

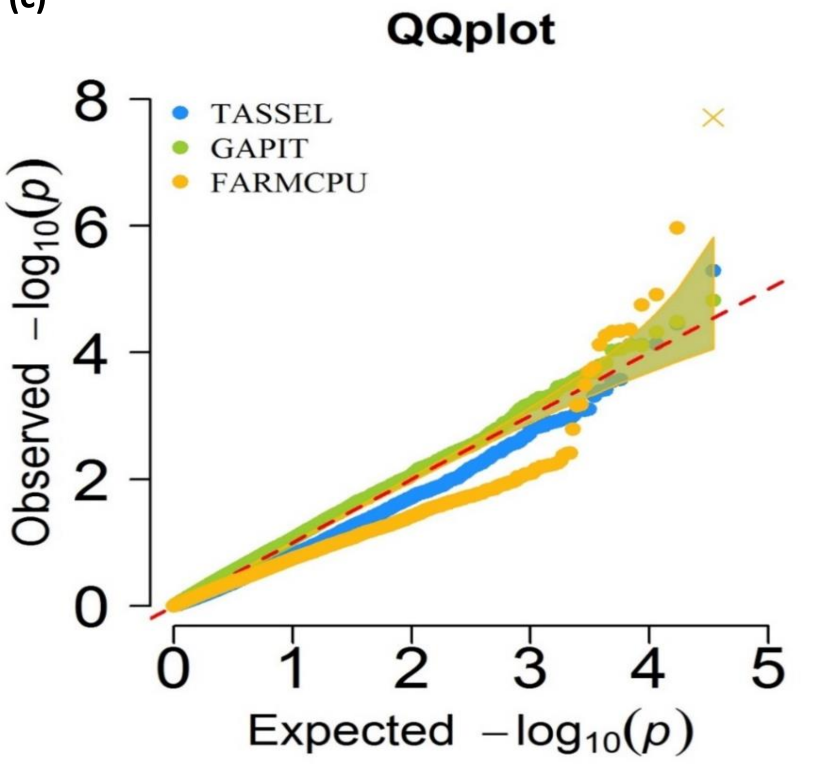
QQplot
TASSEL
GAPIT
FARMCPU
Observed −log10(p)
Expected −log10(p)
0
2
4
6
8
0
1
2
3
4
5

(d)

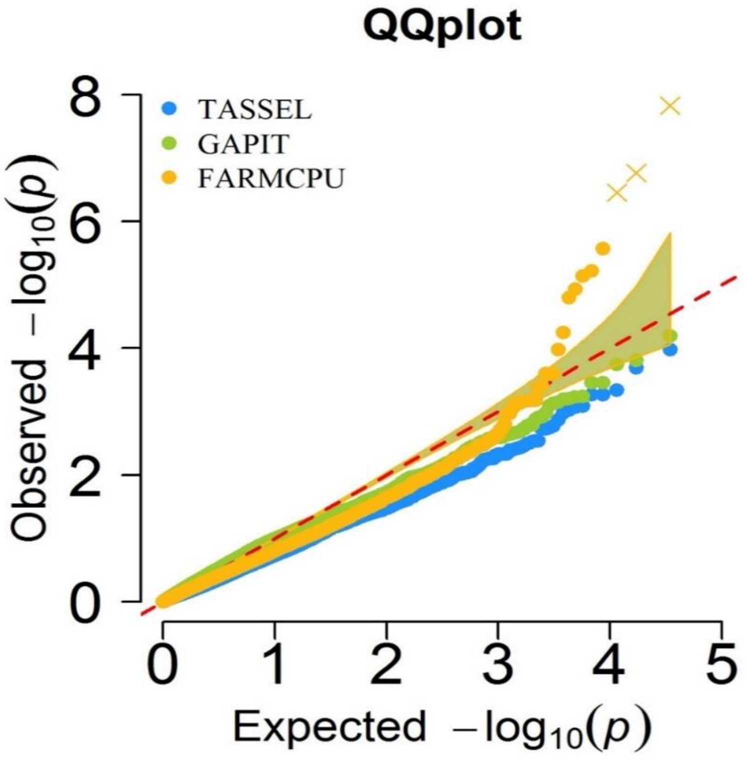
QQplot
TASSEL
GAPIT
FARMCPU
Observed −log10(p)
Expected −log10(p)
0
2
4
6
8
0
1
2
3
4
5

(e)

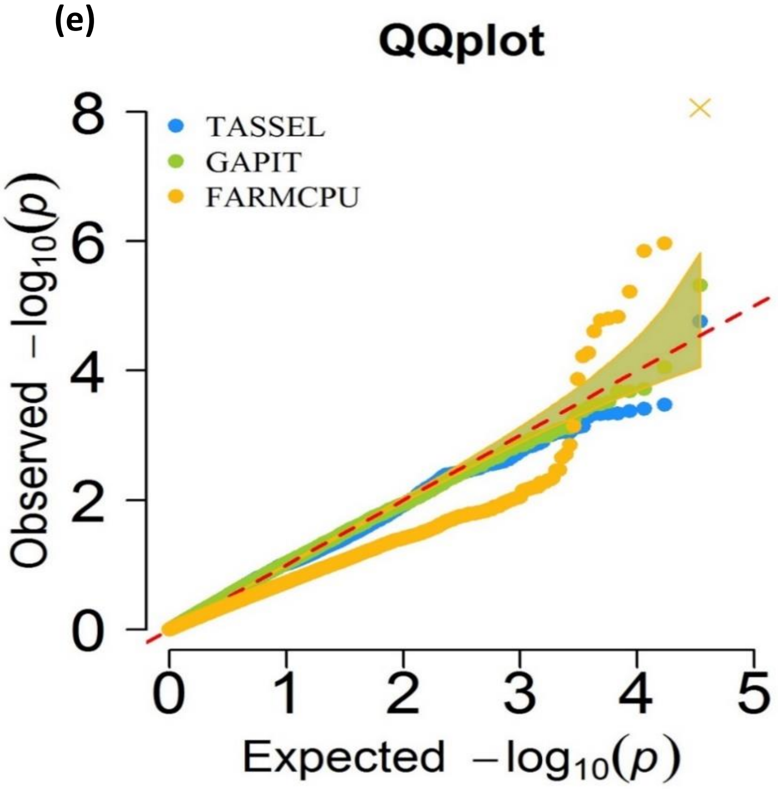

Figure S3: QQ-plots visualize the false positives of three used models based on observed and the expected p values of the GWAS models for (a) relative virus titer, (b) relative total grain weight (c) relative plant height, (d) relative number of ears per plant, (e) relative thousand grain weight.
